# The effects of growth on structural properties of the Achilles and Patellar tendons: A cross‐sectional study

**DOI:** 10.14814/phy2.14544

**Published:** 2020-08-18

**Authors:** Yasuyoshi Mogi

**Affiliations:** ^1^ Faculty of Policy Management Department of Human Life Management Shobi University Kawagoe Japan

**Keywords:** Achilles tendon, adolescent growth spurt, growth, Patellar tendon, tendon cross‐sectional area, tendon length

## Abstract

The purpose of this study was to investigate the structural properties (length and cross‐sectional area) of both the Patellar and Achilles tendons at around adolescent growth spurt. One hundred‐twenty children and adolescents participated in this study. Based on estimated age at peak height velocity, the participants were separated into three groups (before takeoff of adolescent growth spurt group, from takeoff of adolescent growth spurt until peak height velocity group and after peak height velocity group). An ultrasonography technique was used to determine structural properties of the Patellar and Achilles tendons. Significant group difference was observed in tendon length for the Patellar and Achilles tendons among groups. However, there were no significant differences in the ratio of the Patellar tendon to upper leg length and the ratio of the Achilles tendon to lower leg length among groups. The cross‐sectional area of all regions for the Patellar and Achilles tendons in adolescents with after takeoff adolescent growth spurt group was greater than those of before takeoff adolescent growth spurt group. These results indicate that the cross‐sectional area of both the Patellar and Achilles tendons increase with takeoff of adolescent growth spurt and tendons lengthen without the changes in the ratio of tendon length to bone length. In addition, the increases in the cross‐sectional area of both the Patellar and Achilles tendons occur in whole regions but not specific regions.

## Graphical abstract

Tendon cross‐sectional area increases with takeoff of adolescent growth spurt. Tendon cross‐sectional area increases in whole regions but not specific regions.

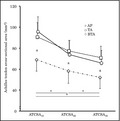

**Abbreviations**
ANOVA: Analysis of variance
AP: After PHV group
ATCSA_10_: The Achilles tendon cross‐sectional area at 10% of the length of the Achilles tendon length from the Achilles tendon insertion
ATCSA_20_: The Achilles tendon cross‐sectional area at 20% of the length of the Achilles tendon length from the Achilles tendon insertion
ATCSA_30_: The Achilles tendon cross‐sectional area at 30% of the length of the Achilles tendon length from the Achilles tendon insertion
BTA: Before takeoff of adolescent growth spurt until PHV group
CV: The coefficient of variation
PHV: Peak height velocity
PTCSA_30_: The Patellar tendon cross‐sectional area at 30% of the Patellar tendon length
PTCSA_50_: The Patellar tendon cross‐sectional area at 50% of the Patellar tendon length
PTCSA_70_: The Patellar tendon cross‐sectional area at 70% of the Patellar tendon length
RMSE: Root mean square error
SD: Standard deviation
TA: From takeoff of adolescent growth spurt until PHV group

## 1 INTRODUCTION

Growth curve of human body including musculoskeletal system has human specific phenomenon called “adolescent growth spurt” (Malina, Bouchard, & Bar‐Or, 2004). Physical characteristics such as stature, body mass, and muscle strength dramatically increase with takeoff of adolescents growth spurt (Tanner, 1981; Lindgren, 1978; Lefevre, Beunen, Steens, Claessens, & Renson, 1990; Kanehisa, Yata, Ikegawa, & Fukunaga, 1995; Kanehisa, Ikegawa, Tsunoda, & Fukunaga, 1995). It is also well known that the “Apophysitis” such as, Sever's disease and Osgood‐Schlatter disease is frequently recognized during adolescent growth spurt (Frisch, Croisier, Urhausen, Seil, & Theisen, 2009; Krivickas, 1997). Many previous studies have been assumed that developing these disorders may be associated with the growth imbalance between bone and muscle‐tendon unit due to adolescent growth spurt (Frisch et al., 2009; Krivickas, 1997; Micheli & Klein, 1991; Micheli & Fehlandt, 1992; Sever, 1912). However, Mogi, Torii, Kawakami, and Yanai (2018) reported that growth imbalance between bone and muscle‐tendon unit was not observed before and after peak height velocity (PHV). Given these finding, therefore, there are conflicting and whether or not the growth imbalance between bone and muscle‐tendon unit is observed during growth period remains unclear. If the muscles and tendons cannot keep up with the longitudinal growth of bones, because of passive tendon stretching due to bone growth, tendon length, and/or the ratio of tendon length to bone length may be greater temporarily in adolescents with takeoff of adolescent growth spurt than those of the others (e.g., before takeoff adolescent growth spurt and/or after PHV). Although it has been attempted previously to investigate the growth changes in structural properties (length and cross‐sectional area) of tendons (Kubo, Teshima, Hirose, & Tsunoda, 2014a, 2014b; O'Brien, Reeves, Baltzopoulos, Jones, & Maganaris, 2010; Neugebauer & Hawkins, 2012; Mersmann et al., 2014, 2017; Mogi et al., 2018; Waugh, Blazevich, Fath, & Korff, 2012.), however, there is few information on the structural properties of the tendons at and/or around adolescent growth spurt (Charcharis, Mersmann, Bohm, & Arampatzis, 2019; Mogi et al., 2018; Mogi, 2019; Pentidis et al., 2019), and no studies have simultaneously examined the structural properties of both the Patellar and Achilles tendons at around adolescent growth spurt.

Magnusson and Kjaer (2003) have demonstrated that cross‐sectional area of the Achilles tendon in distal region was greater than that of proximal region, and stated that region‐specific hypertrophy in cross‐sectional area of tendon occurs in response to the habitual loading. Kongsgaard et al. (2007) also reported that the region‐specific hypertrophy in cross‐sectional area of the Patellar tendon was observed after resistance training. Considering these findings, the increases in the body mass and muscle strength due to adolescent growth spurt may cause region‐specific hypertrophy in cross‐sectional area of both the Patellar and Achilles tendons. Although the cross‐sectional and longitudinal studies previously reported that region‐specific hypertrophy of cross‐sectional area was not observed in the Patellar tendon (O'Brien et al., 2010; Mersmann et al., 2017), these studies did not examine the cross‐sectional area of tendon in adolescents at around adolescent growth spurt.

The present study aimed to investigate the structural properties of both the Patellar and Achilles tendons in children and adolescents among before takeoff of adolescent growth spurt, after takeoff of adolescent growth spurt until PHV and after PHV. Knowledge of how tendon structure grows would not only allow understanding the development of the tendon functions with growth but provide information regarding preventing overuse injuries, such as Osgood‐Schlatter disease and Sever’ disease.

## 2 MATERIALS AND METHODS

### 2.1 Subjects

One hundred‐twenty Japanese children and adolescents aged 6.2–17.9 voluntarily participated in this study. All participants were healthy and had no disability and/or disorder in their lower extremities. Maturity offset (Mirwald, Baxter‐Jones, Bailey, & Beunen, 2002) was used to estimate the age at PHV for participants. Previous studies subjected to Japanese children and adolescents reported that mean age at takeoff of adolescent growth spurt was 10.25 and mean age at PHV was 13.05 (Suwa, Tachibana, Maesaka, Tanaka, & Yokoya, 1992). Based on this study, −2.8 PHV age (the difference between 10.25 and 13.05) was defined as the age at takeoff of adolescent growth spurt in the present study. During the growth period, biological maturation (e.g., PHV age) of children and adolescents varies even in same chorological age. Therefore, PHV age seems to be more accurate than chronological age for detecting the effects of growth on the structural properties of tendons. Thus, the participants whose estimated age at PHV was younger than −2.8 were classified as before takeoff of adolescent growth spurt group (BTA: *n* = 30), the participants whose estimated age at PHV was older than −2.8 and younger than 0 were classified as from takeoff of adolescent growth spurt until PHV group (TA: *n* = 48) and the others were classified as after PHV group (AP: *n* = 42). Anthropometric data of each group were described in Table 1. Prior to the experiments, the purposes of this study and the possible risks associated with the measurements were explained to the participants and their parents, and the written informed consent was obtained from both the participants and the parents. The institutional Human Research Ethics Committee of Japan Society of Human Growth and Development approved this study.

**TABLE 1:** Anthropometric data of all groups (mean ± *SD*)

	BTA	TA	AP
Age (years)	9.1 ± 1.4	12.8 ± 0.7	16.8 ± 0.6
Body height (m)	1.29 ± 0.07	1.51 ± 0.07	1.70 ± 0.06
Body mass (kg)	27.4 ± 4.7	42.3 ± 7.5	60.8 ± 7.6
Upper leg length (mm)	293 ± 24	354 ± 21	390 ± 17
Lower leg length (mm)	294 ± 24	355 ± 22	391 ± 17
Maturity offset	−4.2 ± 0.8	−1.6 ± 0.6	1.7 ± 0.6

Abbreviations: BTA, before takeoff adolescent growth spurt group; TA, from takeoff of adolescent growth spurt until peak height velocity group; AP, after peak height velocity group.

### 2.2 Experimental set‐up

Brightness‐mode (B‐mode) ultrasonography technique was used to measure the length and cross‐sectional area of both the Patellar and Achilles tendons. Transverse and longitudinal ultrasound images were acquired by using ultrasound apparatus (HS‐2000, HONDA ELECTRONICS) with an electronic linear array probe (HLS‐475, HONDA ELECTRONICS). A gel pad was placed between dermal surface and a linear array probe so that the ultrasound images of cross‐sectional area of the tendons were clearly visualized. Ultrasound images were recorded on a Secure Digital memory card through the analog‐to‐digital recorder (GV‐VCBOX, I‐O DATA). Each participant was instructed to relax to avoid any muscle contraction during all measurements.

### 2.3 Measurements and analyses of structural properties of the Patellar tendon

**FIGURE 1:**
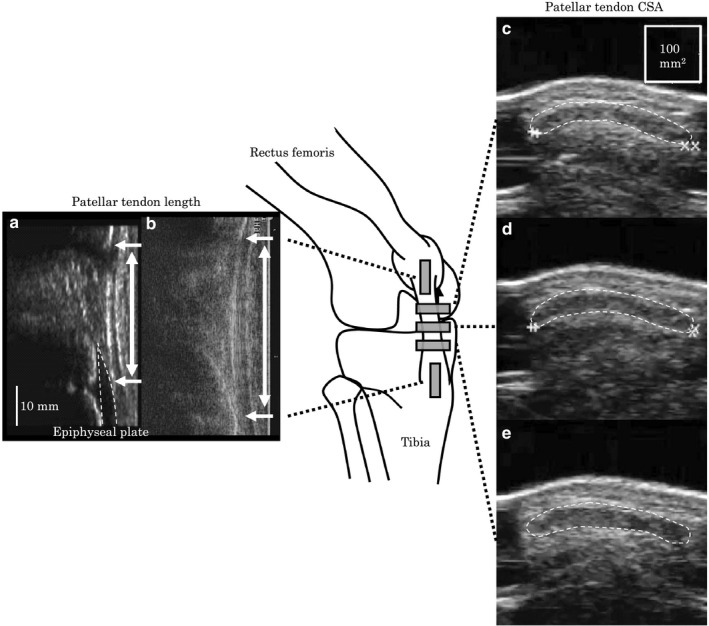
Ultrasound images show the position of lower end of the Patellar tendon and deep insertion of the tendon for 9‐year‐old subject (a) and 17‐year‐old subject (b), and cross‐sectional area (CSA) of the Patellar tendon at 30% (c), 50% (d), and 70% (e) of the Patellar tendon length, respectively. The Patellar tendon is represented by dotted lines

Both the length and cross‐sectional area of the Patellar tendon at rest were measured in a sitting position with the knee joint flexed at an angle of 90 degrees (full extension = 0 degrees). The position of lower end of the Patellar tendon and deep insertion of the tendon on the tibia were detected with B‐mode ultrasonography technique (Figure 1b), and were marked with a pen on the skin surface. In children and adolescents who have epiphyseal plate, the insertion of Patellar tendon was not tibia but epiphyseal plate (Figure 1a). In this case, based on previous study (Kubo et al., 2014b), the insertion of Patellar tendon was determined as the contact point between the lower end of the Patellar tendon and epiphyseal plate instead of deep insertion of the tendon on the tibia. The distance between the two marks was measured along dermal surface and defined as the Patellar tendon length atrest.The distance between the two landmarks was measured along the skin surface by using a flexible measuring tape. The upper leg length was used as an index of femur bone length and measured as the distance between the greater trochanter and popliteal crease. The ratio of Patellar tendon length to upper leg length was calculated. The cross‐sectional area of the Patellar tendon was measured from a transvers ultrasonic image taken at 30% (PTCSA_30_: Figure 1c), 50% (PTCSA_50_: Figure 1d) and 70% (PTCSA_70_: Figure 1e) of the Patellar tendon length. The outline of the tendon in transverse ultrasound images was traced by using software (ImageJ 1.36b, National Institutes of Health), and determined the tendon cross‐sectional area. Tracing was repeated twice, with mean being used for further analysis. These ultrasonography techniques are widely used for measurements of length and cross‐sectional area of the Patellar tendon (O'Brien et al., 2010; Kubo et al., 2014b). In the present study, the repeatability of measurements of Patellar tendon length and cross‐sectional area was investigated on 2 separate days in a preliminary study with 5 children aged 9.4–14.5. There were no significant differences between the test and retest value of the Patellar tendon length and cross‐sectional area. The coefficient variation (CV) was 3.1 ± 1.9% for Patellar tendon length, 3.7 ± 2.7% for PTCSA_30_, 2.9 ± 2.2% for PTCSA_50_ and 1.8 ± 1.5 for PTCSA_70_. The between‐day errors (RMSE) were 1.9 mm for Patellar tendon length, 4.0 mm^2^ for PTCSA_30_, 4.2 mm^2^ for PTCSA_50_ and 2.6 mm^2^ for PTCSA_70_.

### 2.4 Measurements and analyses of structural properties of the Achilles tendon

**FIGURE 2:**
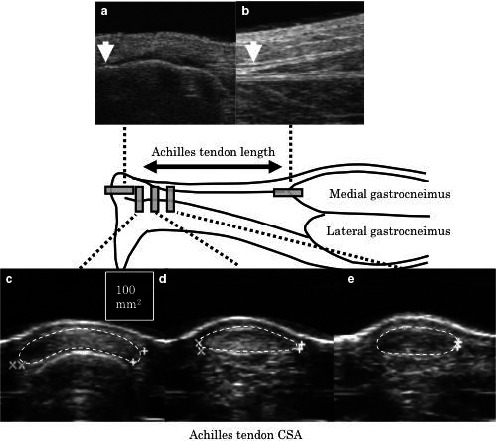
Ultrasound images show the most distal Achilles tendon insertion to the calcaneus (a) and the most distal myotendinous junction of the medial gastrocnemius (b), and cross‐sectional area (CSA) of the Achilles tendon at 10% (c), 20% (d), and 30% (e) of the Achilles tendon length from the Achilles tendon insertion, respectively. The Achilles tendon is represented by dotted lines

Both the length and cross‐sectional area of the Achilles tendon at rest were measured in a prone position with the ankle joint at 90° and the knee fully extended. The positions of the Achilles tendon insertion (Figure 2a) and the distal myotendinous junction of the medial gastrocnemius (Figure 2b) were detected with B‐mode ultrasonography technique and were marked with a pen on the skin surface. The distance between the two marks was measured along the skin surface by using a flexible measuring tape and defined as the Achilles tendon length at rest. The lower leg length was used as an index of tibia bone length and measured as the distance between the popliteal crease and the center of malleolus lateralis. The ratio of Achilles tendon length to lower leg length was calculated. The cross‐sectional area of the Achilles tendon was measured from a transverse ultrasonic image taken at 10% (ATCSA_10_: Figure 2c), 20% (ATCSA_20_: Figure 2d) and 30% (ATCSA_30_: Figure 2e) of the length of the Achilles tendon length from the Achilles tendon insertion. The software and maneuver of tracing for the analysis of Achilles tendon cross‐sectional area were the same as for determining Patellar tendon cross‐sectional area. These ultrasonography techniques are widely used a measurements of length and cross‐sectional area of the Achilles tendon (Kubo et al., 2014a; Mogi et al., 2018). In the present study, the repeatability of measurements of Achilles tendon length and cross‐sectional area was investigated on 2 separate days in a preliminary study with 5 children aged 9.4–14.5. There were no significant differences between the test and retest value of Achilles tendon length and cross‐sectional area. The CV was 0.6 ± 0.6% for Achilles tendon length, 2.5 ± 3.1% for ATCSA_10_, 2.3 ± 1.3% for ATCSA_20_, and 2.7 ± 1.9 for ATCSA_30_. The RMSE were 2.5 mm for Achilles tendon length, 4.8 mm^2^ for ATCSA_10_, 2.8 mm^2^ for ATCSA_20_, and 2.7 mm^2^ for ATCSA_30_.

### 2.5 Statistics

Descriptive data were presented as means and standard deviation (*SD*). A one‐way analysis of variance (ANOVA) was used for Patellar tendon length, ratio of Patellar tendon length to upper leg length, Achilles tendon length and ratio of Achilles tendon length to lower leg length to compare among the 3 groups. If the *F* statistic of the analysis of variance was significant, differences between groups were assessed by a Bonferroni post hoc test. Two‐way ANOVA (group × region) with repeated measures was used to test the effects of the group and region on the cross‐sectional area of the Patellar tendon and the Achilles tendon. If significant interaction was found, multiple comparisons with Bonferroni were conducted to detect simple main effects of groups and regions. The level of statistical significance was set at *p* < .05. Effect size was calculated as η^2^ or partial η^2^. All statistical analyses were conducted by using software (SPSS 25.0J, SPSS Japan). In addition, the statistical power (1 − β) was calculated using software (G* Power 3.1, Faul, Erdfelder, Buchner, & Lang, 2009).

## 3 RESULTS

The results for Patellar tendon length, ratio of Patellar tendon length to upper leg length, Achilles tendon length, and ratio of Achilles tendon length to lower leg length, are shown in Table 2. Significant main effect (group) was found in Patellar tendon length (*p* < .01, η^2^ = 0.47, 1 − β > 0.8) and Achilles tendon length (*p* < .01, η^2^ = 0.47, 1 − β > 0.8). Subsequent analyses revealed that the Patellar tendon length and the Achilles tendon length were significantly greater in AP than the other groups (*p* < .01) and smaller in BTA than TA (*p* < .01). The ratio of Patellar tendon length to upper leg length and ratio of Achilles tendon length to lower leg length were not significantly different among three groups.

**TABLE 2:** Measurement values of all groups (mean ± *SD*)

	BTA	TA	AP	Significance
Patellar tendon length (mm)	31.0 ± 4.5	38.1 ± 5.4	43.1 ± 4.7	*, †
Ratio of Patellar tendon length to upper leg length (%)	10.5 ± 1.1	10.7 ± 1.4	11.0 ± 1.1	n.s.
Achilles tendon length (mm)	151.8 ± 20.7	184.5 ± 16.8	197.5 ± 20.3	*, †
Ratio of Achilles tendon length to lower leg length (%)	51.6 ± 5.9	51.9 ± 4.5	50.4 ± 4.6	n.s.

* Significant difference between BTA (before takeoff adolescent growth spurt group) and the other groups (*p* < .01).

^†^ significant difference between TA (from takeoff of adolescent growth spurt until peak height velocity group) and AP (after peak height velocity group) (*p* < .05).

**FIGURE 3:**
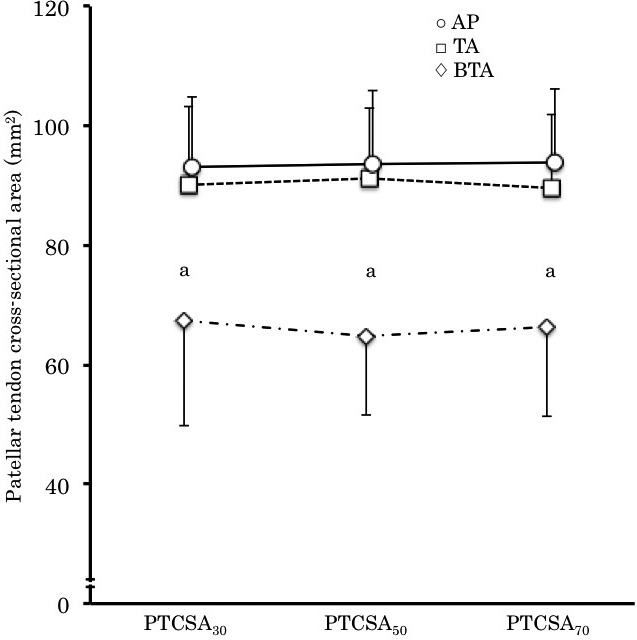
Cross‐sectional area of the Patellar tendon. The circles, the square and the rhombuses represent AP, TA, and BTA, respectively. (a) Significant difference between BTA and the other groups (*p* < .01)

For the cross‐sectional area of the Patellar tendon, two‐way ANOVA revealed that no significant interaction (group × region) was found in the cross‐sectional area of the tendon. However, significant main effect (group) was found (*p* < .01, partial η^2^ = 0.46, 1 − β > 0.8) and the cross‐sectional area of the Patellar tendon was significantly smaller in BTA than those of the other groups (*p* < .01) (Figure 3).

**FIGURE 4:**
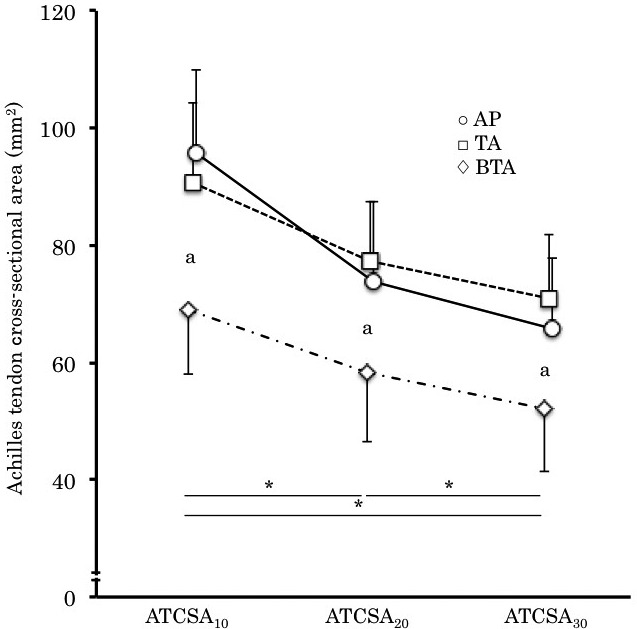
Cross‐sectional area of the Achilles tendon. The circles, the square and the rhombuses represent AP, TA, and BTA, respectively. (*) Significant difference among the regions (*p* < .01). (a) significant difference between BTA and the other groups (*p* < .01)

For the cross‐sectional area of the Achilles tendon, two‐way ANOVA revealed that significant interaction (group × region) was found in the cross‐sectional area of the tendon (*p* < .01, partial η^2^ = 0.12, 1 − β > 0.8). Multiple comparisons showed that the cross‐sectional area of the tendon was significantly smaller in BTA than those of the other groups (*p* < .01) (Figure 4). In addition, the cross‐sectional area of the tendon was greater in ATCSA_10_ than the other regions (*p* < .01), and greater in ATCSA_20_ than that of ATCSA_30_ (*p* < .01) (Figure 4).

## 4 DISCUSSION

The main findings of the present study were that the cross‐sectional area of the Patellar tendon in AP and TA greater than that of BTA. In addition, the cross‐sectional area of the Achilles tendon differed among regions in all groups and there were no regional differences in cross‐sectional area of the Patellar tendon in all groups. Moreover the ratio of tendon length to bone length did not different in the Patellar and Achilles tendons among all groups. These findings indicate that the cross‐sectional area of both the Patellar and Achilles tendons dramatically increases with takeoff of adolescent growth spurt and the increases in cross‐sectional area of both the Patellar and Achilles tendons occur in whole regions but not specific regions, and tendon length may adapt to longitudinal growth of bone adequately even in the period of adolescent growth spurt.

Current results showed for the first time that an increase in the cross‐sectional area of the Patellar tendon occurs with takeoff the adolescent growth spurt. O'Brien et al. (2010) reported that adults have larger Patellar tendon cross‐sectional area than children. Mersmann et al. (2017) also showed that an increase in cross‐sectional area of Patellar tendon in adolescents from 16 years to 18 years old. However, they did not describe the cross‐sectional area of the Patellar tendon at around takeoff adolescent growth spurt. Present results showed that cross‐sectional area of the Patellar tendon did not increase linearly from before takeoff of adolescent growth spurt to after PHV, but it grew dramatically with takeoff of adolescent growth spurt. In addition, the timing of an increase in cross‐sectional area of the Achilles tendon was also observed with takeoff off adolescent growth spurt. Theses results suggest that cross‐sectional area of both the Patellar and Achilles tendons increases at the same time with takeoff of adolescent growth spurt and the growth pattern of the cross‐sectional area of the tendons corresponds to the general type of postnatal growth (Scammon, 1930).

For TA and AP groups, the regions where were measured the Achilles tendon cross‐sectional area in the present study approximately corresponded to 20, 40 and 60 mm regions from calcaneus, respectively. Previous study showed that the cross‐sectional area of Achilles tendon in non‐athlete adults was ≈86.4 mm^2^ at 20 mm, ≈69.4 mm^2^ at 40 mm, and ≈67.8 mm^2^ at 60 mm (These values were calculated by author using the Figure 2 of Magnusson and Kjaer (2003). These values were similar to mean values of cross‐sectional area in TA and AP groups. In addition, the mean values of the cross‐sectional area of the Achilles tendon in BTA were similar with the elementary school children (Kubo et al., 2014a). These results suggest that the cross‐sectional area of the Achilles tendon already reached to that of adults after takeoff of adolescent growth spurt, and support previous results that Achilles tendon cross‐sectional area in junior high school student did not differ that of adults (Kubo et al., 2014a).

The reason why the increase in cross‐sectional area of both the Patellar and Achilles tendons may be explained by the increases in the body mass, muscle strength and hormone secretion with takeoff of adolescent growth spurt (Lindgren, 1978; Lefevre et al., 1990; Malina et al., 2004). From a biomechanical perspective, the increases in the body mass and muscle strength may lead to an increase in mechanical load on the tendons. Previous studies have reported that mechanical load on the tendons would lead to an increase in the tendon metabolism (Evanko & Vogel, 1993; Kjaer et al., 2005; Robbins, Evanko, & Vogel, 1997) and that growth hormone and IGF‐1 also affects the tendon metabolism (Costa et al., 2006; Oliva et al., 2016). Therefore, current results suggest that the tendons hypertrophy in response to the increases in the hormone secretion and mechanical load derived from development of body mass and muscle strength due to takeoff of adolescent growth spurt. Magnusson et al. (2003) stated that the greater tendon size combines to lower the stress on the tendon, which may reduce the risk of injury to the tendon. Thus, it seems reasonable to suppose that the increases in the cross‐sectional area of tendons contribute preservation of the tendon integrity and susceptibility to tendon injuries.

For the Achilles tendon, the cross‐sectional area of distal portion was greater than proximal portion in all groups and the cross‐sectional area greater in TA and AP than BTA in all regions. In addition, cross‐sectional area of the Patellar tendon did not differ between regions within any group, but the cross‐sectional area was greater in TA and AP than BTA in all regions. These results were similar to the previous findings (Magnusson & Kjaer, 2003; O'Brien et al., 2010). Current results suggest that region‐specific hypertrophy in cross‐sectional area of both the Patellar and Achilles tendons is not observed before and after adolescent growth spurt, and that during growth spurt tendon hypertrophies throughout its entire length. Therefore, the assumption that the changes in physical characteristics due to adolescent growth spurt may cause the region‐specific hypertrophy in cross‐sectional area of the Patellar and Achilles tendons was not supported. Hansen et al. (2017) reported that the stress concentrates the region where the minimum tendon cross‐sectional area. Taken this into consideration, if the tendons hypertrophy only at specific regions, more stress derived from muscle force transmission would concentrate on non‐hypertrophic region and these may cause the tendon injuries. Therefore, it is physiologically reasonable that the cross‐sectional area of the tendons increases throughout its entire length so as to reduce the risk of the tendon injuries.

According to the previous findings (Frisch et al., 2009; Krivickas, 1997; Micheli & Klein, 1991; Micheli & Fehlandt, 1992; Sever, 1912), it is assumed that muscle‐tendon unit is passively stretched due to longitudinal growth of bone, especially during adolescent growth spurt. If muscle‐tendon unit cannot keep up with the longitudinal growth of bone, the tendon length in adolescents with takeoff of adolescent growth spurt (TA group) may be greater than the others and/or the ratio of the tendon length to the bone length may differ among groups. However, current results showed that both Patellar and Achilles tendons lengthen and the ratio of tendon length to bone length keeps constant rate in all groups. These results suggest that muscle‐tendon unit may adapt to the longitudinal growth of bone and passive stretching of tendon may not be observed even in a period of adolescent growth spurt. Thus, longitudinal growth of bone does not seem to one of etiological factors for Apophysitis, and the other factors such as overuse (Madden & Mellion, 1996), anatomical malalignment (Scharfbillig, Jones, & Scutter, 2011) and playing surface (Micheli & Klein, 1991; Arnason, Gudmundsson, Dahl, & Jóhannsson, 1996) might be major factors.

There are limitations in the present study. First, present study was a cross‐sectional study and it did not detect within‐subjects changes in the structural properties of both the Patellar and Achilles tendons. Therefore, present results may reflect the underlying properties of individual. Thus, a longitudinal study is needed to confirm the current findings. Second, present study defined Achilles tendon length as the distance between Achilles tendon insertion and the distal myotendinous junction of the medial gastrocnemius, and did not measure the free tendon length. Therefore, if the Achilles tendon length was defined as free tendon length, the present results might be changed. However, our unpublished experimental data (*n* = 54) shows that free tendon length positively correlates to the Achilles tendon length (definition is same as present study). These results indicate that even if the Achilles tendon length is defined as gastrocnemius medialis tendon length, our main findings that tendon lengthens without the changes in the ratio of tendon length to bone length are still reliable. Third, the cross‐sectional area of the Achilles tendon was measured at 10%, 20% and 30% of the Achilles tendon length in the present study. Therefore, present results may not apply to the changes in size of the aponeurosis in the Achilles tendon with growth. The last limitation is associated to the accuracy of measurement of tendon cross‐sectional area using ultrasound method. Recently, previous studies reported that the tendon cross‐sectional area measured with the ultrasound method was underestimated compared to that measured with MRI method (Bohm, Mersmann, Schroll, Mäkitalo, & Arampatzis, 2016; Ekizos et al., 2013; Kruse, Stafilidis, & Tilp, 2017). Bohm et al. (2016) showed that ultrasound method systematically underestimated (19%) the tendon cross‐sectional area values obtained by MRI method. Accordingly, the values of cross‐sectional area of both Achilles and Patellar tendons were re‐calculate by corrected tendon cross‐sectional area that is add 19% and were re‐compared between groups within region and between regions within group so as to examine the effects of this systematic underestimation on the present results. As a result, however, group and region differences in both Achilles and Patellar tendons were still unchanged. Therefore, even if cross‐sectional areas were underestimated due to methodological problem, main findings of present study would not have changed. In addition, the results of present pilot study showed that the repeatability of measurement using ultrasound method was sufficient. These results indicated that the precision of measurement using ultrasound method was found to be reasonable to detect the group and region differences. However, further analysis using MRI methods may be needed to confirm the true values of tendon cross‐sectional area.

## 5 CONCLUSIONS

In conclusion, the cross‐sectional area of both the Patellar and Achilles tendons increases with takeoff of adolescent growth spurt and the tendons lengthen without the changes in the ratio of tendon length to bone length. In addition, the increases in the cross‐sectional area of both the Patellar and Achilles tendons occur in whole regions but not specific regions.

## CONFLICT OF INTEREST

The author has no conflict of interest.

## AUTHOR CONTRIBUTIONS

YM participated in study design, data collection and analysis, and drafted manuscript.
